# Age-dependent mechanisms of exercise in the treatment of depression: a comprehensive review of physiological and psychological pathways

**DOI:** 10.3389/fpsyg.2025.1562434

**Published:** 2025-04-17

**Authors:** Peng Xue, Xingbin Du, Jianda Kong

**Affiliations:** ^1^College of General Education, Shandong Huayu University of Technology, Dezhou, China; ^2^College of Sports Science, Qufu Normal University, Qufu, China

**Keywords:** exercise, depression, treatment, age-dependency, mechanisms

## Abstract

Depression has become one of the most common mental disorders in the world. The rising incidence rate and disability rate pose a serious challenge to public health and socio-economic development. Traditional medication and psychotherapy are positive, but they often come with limitations such as side effects, poor compliance, and resource constraints, which highlights the urgent need for more proactive and sustainable non pharmacological interventions. We mainly explored the physiological and psychological mechanisms of exercise in alleviating depression in different age groups. In particular, we evaluated the characteristics and influencing factors of depression in each age group and compared the pathways through which exercise works, aiming to provide scientific basis for clinical practice and public health policies, and strengthen the application of non pharmacological treatment in depression management. It is worth noting that, in the context of a comprehensive search and analysis of recent literature, we have covered the epidemiology of depression, the impact of exercise on mental health, the characteristics of depression in different age groups, and the specific ways in which exercise alleviates depression through physiological and psychological mechanisms. Exercise alleviates symptoms of depression by regulating neurotransmitters, enhancing neuroplasticity, regulating hormone levels, reducing inflammatory responses through physiological pathways, as well as enhancing cognitive function, strengthening emotional regulation, triggering social interactions, and improving self-efficacy through psychological pathways. The differences in physiological and psychological mechanisms among different age groups determine the age dependent characteristics of exercise in mitigating depression. Teenagers, middle-aged people, and elderly people can improve depressive symptoms by enhancing neural progression, regulating stress responses, and strengthening social support, respectively.

## 1 Introduction

Depression, globally, as one of the most prevalent mental disorders has exhibited a significant upward tendency in both incidence and disability rates, posing a severe challenge to public health. In the context of reports by the World Health Organization (WHO), depression has turned into one of the leading causes of disability worldwide, affecting the quality of life of hundreds of millions of people, and hindering socioeconomic progression (Ferrari et al., 2013). Conventional treatment methods for depression mainly count on pharmacotherapy and psychotherapy; nonetheless, these strategies have certain limitations, such as medication side effects, poor treatment adherence, and inadequate resources for psychological therapies (Gabriel et al., 2020). Consequently, investigating positive and sustainable non-pharmacological treatment modalities has turned into a critical focus of current research.

Exercise has been proven to have enormous potential in preventing and treating depression. Numerous studies have confirmed that regular exercise not only improves an individual’s physical health, but also enhances their mental wellbeing. Exercise can alleviate symptoms of depression through various physiological and psychological mechanisms, enhance the regulation of neurotransmitters in the brain, improve cognitive function, enhance emotional regulation, and promote social interaction. However, the differences in physiological and psychological progression stages among individuals of various age groups have led to significant age-related characteristics in the mechanism and effectiveness of exercise therapy for depression (Miao et al., 2024). Specifically, during adolescence, exercise significantly affects brain development and emotional stability. Middle aged people face dual pressures of career and family, and benefit from exercise by regulating stress hormones and enhancing cardiovascular health, thereby slowing depression symptoms caused by stress. In addition, exercise plays a crucial role in preventing cognitive decline and enhancing social interaction among the elderly population, as physiological decline and reduced social support mainly involve the psychological and practical help individuals receive through interactions within social networks. Strong social support is associated with reducing loneliness, enhancing emotional resilience, and improving psychological outcomes, particularly in the management of depression (Sutton et al., 2022).

Therefore, our review aims to explore in depth the physiological and psychological mechanisms by which exercise alleviates depression in various age groups. We analyzed the characteristics and influencing factors of depression in various age groups, and discussed in detail the mechanism by which exercise alleviates depression through physiological pathways, in order to compare the differences in physiological responses among various age groups. We also explored the mechanism by which exercise affects depression through psychological pathways. We comprehensively propose multiple mechanisms for exercise to alleviate depression in various age groups, providing scientific basis for clinical practice and public health policies, and strengthening the widespread application of non pharmacological treatment methods in depression management.

## 2 Overview of depression and exercise

### 2.1 Definition and diagnosis of depression

Depression is a mental disorder defined by persistent low mood, loss of interest, lowered energy, and reduced self-esteem (Kessler and Bromet, 2013). In the context of the Diagnostic and Statistical Manual of Mental Disorders, Fifth Edition (DSM-5), the diagnostic criteria for depression, include the presence of major depressive symptoms for at least 2 weeks, with these symptoms can significantly affecting the individual’s daily functioning (American Psychiatric Association, 2013). Besides, depression manifests in various forms, including various types, such as unipolar depression, recurrent depression, and bipolar depression (Ostacher, 2018). Epidemiological data, globally, indicate that the prevalence of depression is increasingly enhanced, making it a significant public health concern (Depression, 2017). According to the WHO, depression has become one of the leading factors of disability worldwide, uncovered vitally affecting individuals’ quality of life and socioeconomic progression (Ferrari et al., 2013).

### 2.2 Overall impact of exercise on mental health

#### 2.2.1 Mood improvement

Exercise promotes mood improvement through various mechanisms, as well as stimulates the brain to produce endorphins, in detail, a class of natural analgesics that enhance mood and alleviate anxiety and depressive feelings (Meeusen and De Meirleir, 1995). Besides, Dishman’s monograph confirms that exercise boosts the release of neurotransmitters, such as serotonin and dopamine, which play crucial roles in regulating mood and emotions (Dishman et al., 2021). Furthermore, exercise, apparently, helps individuals divert their attention, reducing the focus on negative emotions, hence positively mitigating depressive symptoms (Craft and Perna, 2004). Evidence has suggested that regular aerobic exercises, such as running, swimming, and cycling, can significantly decline the incidence of depression and enhance overall mood states (Schuch et al., 2016). Notably, substantial body of research demonstrates that regular exercise significantly enhances mental health by slowing depressive symptoms and improving cognitive function (Schuch et al., 2016; Craft and Perna, 2004). Large-scale epidemiological studies confirm that increased physical activity is associated with lower odds of depression, with each additional day of vigorous exercise reducing depressive symptom risk by 11% (Huang and Huang, 2023a). Conversely, higher sedentary time correlates with increased depression prevalence, reinforcing the importance of physical activity as a non-pharmacological intervention for mental health (Huang and Huang, 2023a). Furthermore, it is notably worthy that a systematic review further confirmed that exercise therapy provides therapeutic benefits comparable to traditional pharmacological treatments, particularly when prescribed with moderate intensity and adequate duration (Xie et al., 2021).

#### 2.2.2 Cognitive function enhancement

Exercise has a positive impact on mood, as well as significantly improves cognitive function (Hartwig, 2010). Exercise has been suggested to enhance neural plasticity (as the brain’s ability to reorganize and adapt its structure and function in response to experiences, learning, or environmental changes) in the brain, interestingly, strengthening the generation of new neurons and the formation of neural connections, hence strengthening memory and learning abilities (Kingsbury et al., 2000). Besides, exercise increases cerebral blood flow, triggering the supply of oxygen and nutrients, and enhancing brain metabolic functions (Alomary and Belhadj, 2007). These physiological changes cause improved attention, executive function, and information processing speed, ultimately enhancing cognitive performance (Ratey, 2008). In the elderly population, in particular, regular exercise has been uncovered to delay cognitive decline and prevent the onset of dementia (Brock et al., 2011).

## 3 Features of depression across various age groups

Depression demonstrates significant variations in its manifestations, and impacting factors across different stages of the life cycle. Determining these differences is essential for developing targeted prevention and intervention strategies to enhance treatment efficacy. Table 1 displays features of depression across various age groups.

**Table 1:** Features of depression across various age groups.

Feature	Adolescents	Middle age	Elderly	References
Prevalence (%)	10–20%, with a significantly higher incidence in female adolescents compared to males.	Not specifically provided, but depression is notably prevalent in this age group.	15–30%	Vidal et al. (2020), Blazer et al. (2001)
Gender differences	Female adolescents exhibit a significantly higher incidence of depression than their male counterparts	Not specifically mentioned.	Not specifically mentioned	Vidal et al. (2020)
Depression features	Persistent low mood, Loss of interest, Declining academic performance, and Strained interpersonal relationships.	Emotional fluctuations, Anxiety, and Accumulation of depressive moods.	Persistent sadness, Loss of interest, Decline in physical and cognitive functions. Social isolation, and Excessive preoccupation with death	Miao et al. (2024), Vidal et al. (2020), Blazer et al. (2001), Lin et al. (2024), Li et al. (2021), Arthur et al. (2020), Pocklington (2017)
Primary impacting factors	Academic pressure, Family environment, Social relationships, Gender role identity, Genetic predispositions, Widespread use of the internet and social media leading to decreased self-esteem and increased feelings of loneliness.	Career pressures, Family responsibilities, Financial burdens, Health issues. Role conflicts (e.g., balancing work and family), Time management difficulties, Changes in social roles (e.g., children becoming independent, deterioration of parents’ health), and Hormonal fluctuations (e.g., perimenopausal period).	Chronic diseases (e.g., cardiovascular diseases, diabetes), Deterioration of physical functions Bereavement or loss of close friends and family members, Lack of social support systems, Economic pressures, and Degenerative changes in the brain Neurotransmitter imbalances.	Miao et al. (2024), Vidal et al. (2020), Blazer et al. (2001), Lin et al. (2024), Li et al. (2021), Arthur et al. (2020), Pocklington (2017)
Unique aspects	Increased psychological stress due to internet and social media use, and Decline in self-esteem and increase in feelings of loneliness.	Time management difficulties from balancing work and family, and Psychological stress from changes such as children leaving home or parents’ health deterioration.	Perceptions and attitudes toward depression affect willingness to seek treatment, and Biological causes (e.g., neurotransmitter imbalances) play a crucial role in the pathogenesis of depression.	Miao et al. (2024), Vidal et al. (2020), Blazer et al. (2001), Lin et al. (2024), Li et al. (2021), Arthur et al. (2020), Pocklington (2017)

### 3.1 Adolescents—epidemiological features and primary impacting factors

Adolescence is a critical period for psychological progression and social adaptation, during which the incidence of depression shows an upward trend. According to relevant evidence, the prevalence of depression among adolescents is roughly 10–20%, with notable gender differences; the incidence rate in female adolescents is significantly higher than in males (Vidal et al., 2020). features of depression during adolescence specifically include persistent low mood, loss of interest, declining academic performance—often defined by reduced concentration, impaired memory, difficulties in maintaining motivation, heightened anxiety related to examinations, and increased absenteeism—as well as strained interpersonal relationships an increasing from heightened sensitivity to peer evaluations and familial expectations (Vidal et al., 2020). Academic pressures, apparently, including intense competition, demanding curricula, and high parental or societal expectations for achievement, have been identified as significant stressors causing depressive symptoms in this age group (Vidal et al., 2020). Primary impacting factors, expressly, include academic pressure, family environment, social relationships, gender role identity, and genetic predispositions (Vidal et al., 2020). Furthermore, the widespread use of the internet and social media has heightened psychological stress among adolescents, causing declined self-esteem and increased feelings of loneliness, hence elevating the risk of depression (Vidal et al., 2020).

### 3.2 Middle age—unique challenges and psychosocial context

Depression in middle age presents unique challenges, and is affected by distinct psychosocial contexts. Individuals in middle age typically face multiple stressors, comparising career pressures, family responsibilities, financial burdens, and health issues (Miao et al., 2024). These causes can cause emotional fluctuations, anxiety, and the accumulation of depressive moods (Lin et al., 2024). Besides, research by Li et al., apparently, has uncovered that middle-aged individuals commonly experience role conflicts and difficulties in time management while balancing work and family, which further trigger psychological stress (Li et al., 2021). Changes in social roles, such as children becoming independent and leaving home or the deterioration of parents’ health, can also boost depressive symptoms (Lin et al., 2024). Biologically, it is notable that hormonal fluctuations during middle age (e.g., the perimenopausal period) may affect emotional stability, exacerbating the risk of depression (Miao et al., 2024).

### 3.3 Elderly—epidemiological trends and specific impacting factors

Depression among the elderly, different from that among adolescents and middle ages, is a significant concern that cannot be neglected. In the context of the increasing aging population, the prevalence of depression in older adults, apparently, is gradually increasing, accounting for roughly 15–30% of the elderly populations (Blazer et al., 2001; Arthur et al., 2020). Besides, it is worth noting that features of depression in the elderly, include persistent sadness, loss of interest, decline in physical and cognitive functions, social isolation, and excessive preoccupation with death. Specific impacting factors, mainly include the presence of chronic diseases (such as cardiovascular diseases and diabetes), deterioration of physical functions, bereavement or loss of close friends and family members, lack of social support systems, and economic pressures (Blazer et al., 2001; Pocklington, 2017). Furthermore, the elderly’s perceptions and attitudes toward depression, interestingly, can affect their willingness to seek treatment, causing the worsening of depressive symptoms and lowered treatment efficacy. Biological causes, such as degenerative changes in the brain and imbalances in neurotransmitters, meanwhile, play crucial roles in the pathogenesis of depression in the elderly (Arthur et al., 2020).

## 4 Physiological mechanisms of exercise in slowing depression

**Figure 1:**
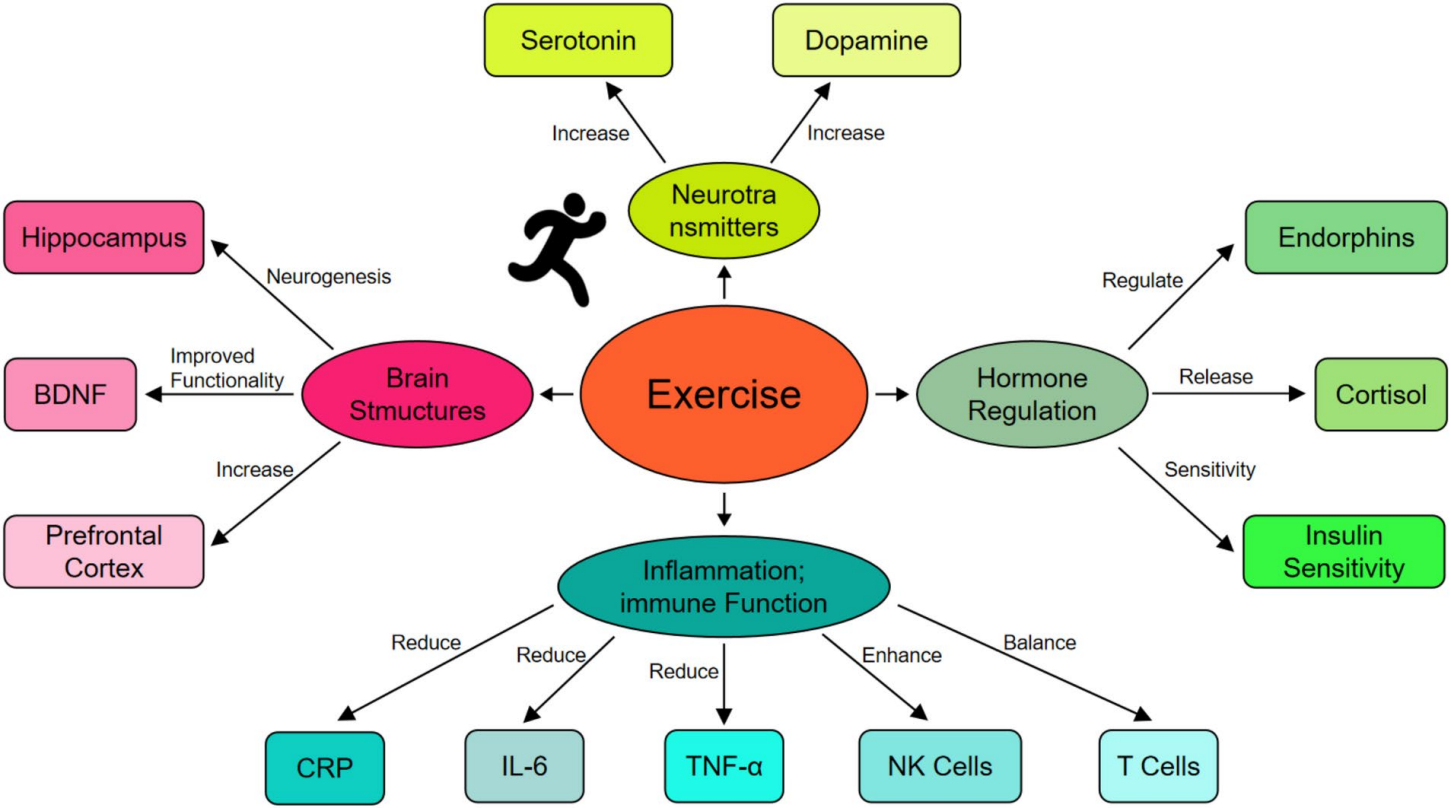
The physiological mechanism of exercise in slowing depression. This figure displays the physiological mechanisms through which exercise alleviates depression. Exercise regulates neurotransmitters, such as serotonin and dopamine, enhancing mood stability and emotional resilience. It also induces positive structural changes in the brain, including improved neuroplasticity in the hippocampus and enhanced functionality of the prefrontal cortex. Additionally, exercise modulates stress hormones by reducing cortisol levels and promoting the release of positive hormones like endorphins, which contribute to a sense of wellbeing. Exercise’s anti-inflammatory effects are evident in the reduction of inflammatory markers (e.g., CRP, IL-6, and TNF-α) and the enhancement of immune functions, such as increased NK cell activity and balanced T-cell responses. These mechanisms collectively mitigate the negative effects of chronic inflammation and stress on the central nervous system, ultimately reducing depressive symptoms and improving mental health.

Exercise plays positive effects on depression through multiple physiological pathways, comparising alterations in neurotransmitters and brain structures, hormone regulation, and modulation of inflammation and immune functions. These mechanisms may exhibit distinct features and effects across various age groups, further affecting the role of exercise in the treatment of depression. Figure 1 exhibits the physiological mechanism of exercise in slowing depression.

### 4.1 Changes in neurotransmitters and brain structures

#### 4.1.1 Regulation of neurotransmitters

Exercise significantly impacts the levels of neurotransmitters in the brain, which play critical roles in regulating mood and emotions. Literature confirms that regular aerobic exercises, such as running, swimming, and cycling facilitate the release of neurotransmitters like serotonin, norepinephrine, and dopamine, changes that cause improved mood and enhanced emotional resilience (Childs and De Wit, 2014). Notably, serotonin is crucial for mood regulation and feelings of wellbeing; exercise increases serotonin activity in the brain, hence facilitating mood enhancement (Heijnen et al., 1890). The elevation of these neurotransmitters helps alleviate anxiety and depressive symptoms, highlighting the significant role of exercise in mental health (Childs and De Wit, 2014). Population-based studies suggest that increased physical activity—particularly vigorous exercise—is significantly correlated with lower depression prevalence, further reinforcing the role of exercise-induced neurobiological adaptations in mitigating depressive symptoms (Huang and Huang, 2023a). Additionally, acute exercise bouts have been shown to transiently elevate brain-derived neurotrophic factor (BDNF) and modulate immuno-inflammatory pathways, potentially causing its antidepressant effects (Ross et al., 2023). However, inconsistencies in long-term exercise training studies highlight the complexity of these mechanisms, underscoring the need for further research into the biological underpinnings of exercise-induced antidepressant effects.

#### 4.1.2 Changes in brain structures

Long-term exercise induces positive changes in brain structures, in particular, in regions tightly linked to emotion regulation and cognitive function, such as the hippocampus and prefrontal cortex. Study evidences, in detail, have displayed that physical exercise promotes neural plasticity, causing increased hippocampal volume and enhanced neural functionality, which in turn improves memory and learning capabilities (Li et al., 2024; Ballesteros et al., 2018). Furthermore, exercise improves blood flow and metabolic activity in the prefrontal cortex, consequently, enhancing its functionality and strengthening individuals’ abilities in emotion regulation and decision-making (Li et al., 2024; Ballesteros et al., 2018).

#### 4.1.3 Enhancement of neuroplasticity

Neuroplasticity enhancement plays a key role in combating depression. Study has suggested that exercise increases the concentration of brain-derived neurotrophic factor (BDNF), which regulates neuroplasticity and partially alleviates depressive symptoms. For instance, existing research confirms that both acute and long-term regular exercise positively elevate BDNF levels, hence strengthening neuronal growth and repair (Jasińska-Mikołajczyk et al., 2022). Besides, BDNF not only plays a significant role within the nervous system, but is also released through muscle contractions, exerting multiple effects on the nervous system. This process is considered to hold substantial potential in the treatment of depression (Murawska-Ciałowicz et al., 2021).

### 4.2 Hormone regulation

#### 4.2.1 Regulation of stress hormones

Exercise plays a crucial role in regulating stress hormone levels, in particular, cortisol. Evidence has suggested that regular exercise can improve the functioning of the hypothalamic–pituitary–adrenal (HPA) axis, positively reducing cortisol secretion levels, specifically, cortisol is the primary stress hormone in the body, and its chronic elevation is tightly linked to mental health issues such as depression (Moyers and Hagger, 2023). Consequently, in the context of regulating cortisol levels via exercise, it is possible to mitigate stress responses and alleviate depression symptoms related to stress. Existing researches confirmed that exercise improves emotional stability through this mechanism, especially by regulating the HPA axis and reducing cortisol levels (Moyers and Hagger, 2023; De Nys et al., 2022).

#### 4.2.2 Release of positive hormones

Exercise not only supperess the secretion of stress hormones, but also promotes the release of positive hormones, such as endorphins and dopamine. Endorphins, natural analgesics, enhance mood and generate feelings of pleasure, shaping the biological basis for the “runner’s high” phenomenon, hence significantly increasing individuals’ sense of wellbeing and satisfaction (Loeb et al., 2022). Studies have suggested that the release of endorphins during exercise may play a key role in strengthening neurogenesis (as the biological process involving the generation of new neurons, particularly in brain regions such as the hippocampus, which is critical for learning, memory, and emotional regulation) in adults and associated behavioral strengths (Schoenfeld and Swanson, 2021; Ando et al., 2024). Furthermore, exercise increases dopamine levels, a “feel-good” hormone essential for the functioning of the brain’s reward system, which enhances motivation and positive behaviors, hence strengthening emotional states (Ando et al., 2024).

#### 4.2.3 Maintenance of hormonal balance

Exercise helps maintain hormonal balance by regulating various hormones, hence supporting the stability of the endocrine system and positively affecting the prevention and alleviation of depression. For example, exercise strengthens insulin sensitivity, improves blood glucose control, and indirectly improves emotional health by reducing the risk of metabolic syndrome (Mennitti et al., 2024). Besides, exercise regulates the levels of sex hormones such as estrogen and testosterone, which in turn affect mood and the manifestation of depressive symptoms. Study confirms that endurance training and high-intensity interval training (HIIT) not only improve insulin resistance, but also facilitate increases in testosterone levels, changes that may help alleviate depressive symptoms (de Souza et al., 2017).

### 4.3 Inflammation and immune function

#### 4.3.1 Association between depression and inflammation

Recent studies have identified a significant association between chronic inflammation and depression (Gavril et al., 2024; Maydych, 2019). Patients with depression typically exhibit elevated levels of inflammatory markers, such as C-reactive protein (CRP), tumor necrosis factor-α (TNF-α), and interleukin-6 (IL-6), which can impact the functioning of the central nervous system, causing mood disorders and cognitive decline (Gavril et al., 2024). Study has suggested that individuals with depression generally display higher levels of interleukin-1 (IL-1), IL-6, TNF-α, and CRP compared to non-depressed individuals (Maydych, 2019). Furthermore, the concentration of inflammatory markers is significantly correlated with the severity of depressive symptoms (Gavril et al., 2024). Interestingly, emerging evidence suggests that nutritional causes may influence depression risk through immune modulation. A machine learning-based analysis of a large-scale cohort identified potassium, vitamin E, and vitamin K intake as significant dietary contributors to depressive symptoms, highlighting potential nutritional interventions for reducing inflammation and improving mental health (Huang and Huang, 2023b). Chronic inflammatory states not only exacerbate depressive symptoms, but may also affect treatment efficacy and prognosis.

#### 4.3.2 Impact of exercise on inflammatory markers

Exercise has been proven to positively reduce levels of inflammatory markers in the body, in particular, CRP, TNF-α, and IL-6, with this anti-inflammatory effect playing a profound role in strengthening immune function (Cerqueira et al., 2020). For example, article has displayed that regular aerobic exercise significantly declines levels of these inflammatory markers in middle-aged and elderly populations (Zheng et al., 2019). Furthermore, exercise supports the repair processes of BDNF, hence strengthening the nervous system’s anti-inflammatory defenses and boosting mood and cognitive functions (Hu et al., 2023).

#### 4.3.3 Regulation of the immune system

Exercise significantly enhances the body’s immune response by regulating immune system functions. Studies confirmed that exercise promotes the circulation of lymphocytes, in particular, natural killer (NK) cells, and enhances their activity, hence increasing the body’s resistance to pathogens (Cerqueira et al., 2020; Gustafson et al., 2021). For instance, acute dynamic exercises, such as running or cycling, can rapidly mobilize white blood cells into the bloodstream, with NK cell numbers increasing three to fivefold during exercise, hence enhancing immune responses to infections and tumors (Cerqueira et al., 2020; Gustafson et al., 2021). Furthermore, moderate exercise helps regulate the secretion functions of immune cells, strengthening the balance of T cells, reducing levels of pro-inflammatory cytokines, and increasing the release of anti-inflammatory cytokines, hence diminishing the occurrence of chronic low-grade inflammation. This immune regulatory effect is particularly significant for reducing the risk of infections and strengthening mental health status in elderly populations (Romeo et al., 2010).

#### 4.3.4 Age-dependent inflammatory and immune responses

Individuals of various age groups exhibit variations in their inflammatory and immune responses. Adolescents, whose immune systems are still developing, may benefit from exercise through the maturation and optimization of immune functions (Brauer et al., 2021). Middle-aged individuals, commonly subjected to long-term work and life-related stress, are typically in a chronic inflammatory state; exercise can slow depressive symptoms by reducing inflammatory responses (Shao et al., 2021). In elderly populations, immune function gradually declines, and exercise can enhance immune responses, decrease chronic inflammation, and prevent immune dysregulation linked to depression (Brauer et al., 2021; Phillips and Fahimi, 2018).

### 4.4 Critical analysis of exercise mechanisms in depression treatment

#### 4.4.1 Variability in exercise-induced neurobiological changes

Exercise is widely acknowledged to influence neurotransmitter systems, neuroplasticity, and inflammatory pathways, all of which are implicated in depression (Black et al., 2015). However, studies have reported heterogeneous findings regarding the extent and consistency of these effects. While acute exercise bouts have been shown to transiently elevate serotonin, norepinephrine, and brain-derived neurotrophic factor (BDNF) levels, long-term exercise training has not consistently displayed sustained changes in these biomarkers. Research suggests that acute exercise positively influences mood and cognition by modulating neurophysiological and neurochemical pathways, including transient increases in BDNF levels (Basso and Suzuki, 2017). However, while long-term exercise has been associated with memory improvements and peripheral BDNF modulation, its effects on sustained biomarker changes remain inconsistent (De la Rosa et al., 2019).

An umbrella review has also reported variability in outcomes, with some studies failing to show a statistically significant improvement in depressive symptoms following exercise interventions (Liu et al., 2025). Methodological differences, including variations in exercise intensity, duration, and participant adherence, contribute to these discrepancies. Research indicates that physical activity is highly beneficial for improving symptoms of depression, anxiety, and distress across a wide range of adult populations positive (Singh et al., 2023). Some studies suggest that the antidepressant effects of exercise may be more pronounced in specific subgroups, such as individuals with mild-to-moderate depression, whereas those with severe depression may require multimodal interventions combining exercise with pharmacotherapy or psychotherapy (Noetel et al., 2024). Additionally, walking or jogging, yoga, and strength training have been found to be particularly positive, especially when performed at higher intensities (Noetel et al., 2024).

#### 4.4.2 Challenges in distinguishing age-dependent mechanisms

Although the mechanisms by which exercise alleviates depression are theorized to vary across life stages, there remains a lack of direct empirical evidence distinguishing these mechanisms among various age groups. Evidence investigates exercise and depression do not stratify participants by age, making it difficult to determine whether observed effects are truly age-dependent or driven by other causes such as baseline fitness, comorbidities, or lifestyle differences (Noetel et al., 2024).

Theoretically, adolescence is a period of heightened neuroplasticity, suggesting that exercise may exert stronger effects on synaptic remodeling and cognitive function in younger populations progression (Larsen and Luna, 2018; Li et al., 2024). Middle-aged adults, experiencing heightened stress and hormonal fluctuations, may benefit from exercise mainly through HPA-axis modulation and cardiovascular health improvements (Lucertini et al., 2015). In contrast, older adults may experience antidepressant benefits through reduced neurodegeneration, improved immune function, and enhanced social interaction (Neațu et al., 2024; Wang et al., 2016). However, direct comparisons between these age groups remain scarce, and meta-analyses have reported no statistically significant differences in the antidepressant effects of exercise across different age cohorts.

## 5 Psychological mechanisms of exercise in slowing depression

Exercise plays positive effects on depression through various psychological pathways, mainly comparising the enhancement of cognitive functions, emotional regulation and stress management, social interaction and support, as well as the improvement of self-efficacy and self-esteem. These psychological mechanisms may exhibit distinct features and effects across various age groups, further affecting the role of exercise in the treatment of depression. Table 2 displays psychological mechanisms through which physical activity alleviates depression.

**Table 2:** Psychological mechanisms through which physical activity alleviates depression.

Psychological mechanism	Mechanism description	Neuro/biological mechanisms	Behavioral changes	Impact on depression	Effects across various age groups	References
Enhancement of cognitive functions	Improves attention, memory, and executive functions.	Increases hippocampal volume, enhances prefrontal cortex functionality, boosts brain plasticity, elevates BDNF levels, and improves oxygenation in relevant brain regions.	Enhances cognitive flexibility, problem-solving abilities, decision-making, and self-control.	Improves emotion management and behavioral patterns, enhances cognitive functions across all age groups, and strengthens the ability to manage negative emotions.	Beneficial for both young adults and elderly populations, with cardiovascular health being significantly linked to executive control in older adults.	Ferrer-Uris et al. (2022), Chaire et al. (2020), Kukla-Bartoszek and Głombik (2024), Mullen and Hall (2015), Buckley et al. (2014), Zhou and Tolmie (2024)
Emotional regulation and stress management	Stabilizes emotions, reduces emotional fluctuations, and enhances stress coping abilities.	Enhances activity in the prefrontal cortex and amygdala, increases cortical thickness of the prefrontal cortex, releases endorphins, lowers cortisol levels, and regulates the HPA axis.	Increases emotional stability, reduces emotional volatility, and strengthens resilience to stress.	Decreases depressive symptoms, lowers anxiety and stress responses, and improves overall psychological health.	Positive effects across all age groups, with long-term participation aiding in sustained stress management and emotional stability.	Moyers and Hagger (2023), Kukla-Bartoszek and Głombik (2024), Lim et al. (2021), Wu et al. (2022), Lin et al. (2024), Gosadi (2024), Zhang et al. (1888), Mu et al. (2024), Bhatt et al. (2024)
Social interaction and support	Promotes social interactions, builds support networks, and reduces social isolation.	Establishes social platforms that enhance social skills and self-concept, and strengthens social support systems.	Increases social activities, reduces feelings of loneliness, and reinforces social support networks.	Alleviates loneliness, reduces depressive symptoms, improves interpersonal relationships, and enhances psychological recovery.	Various age groups build various forms of social support through group activities, with older adults potentially relying more on team activities for support.	Li et al. (2024), Elgendy et al. (2024), Zhang et al. (2022), Liu et al. (2023), Martín-Rodríguez et al. (2024), Blake (2012), Snowden et al. (2015)
Improvement of self-efficacy and self-esteem	Sets and achieves exercise goals, enhances self-efficacy and self-esteem, and boosts confidence and life satisfaction.	Promotes a positive self-image and psychological capital, and increases self-efficacy through goal achievement in physical activities.	Achieves exercise goals, enhances self-control and goal-oriented behaviors, and improves self-image.	Increases confidence, reduces anxiety and depressive emotions, improves interpersonal relationships, and boosts life satisfaction.	Adults and elderly individuals enhance self-efficacy and self-esteem through physical activity, thereby slowing depressive symptoms.	Li et al. (2024), Mu et al. (2024), Wei et al. (2024)

### 5.1 Enhancement of cognitive functions

Exercise has been proven to profoundly improve individuals’ cognitive functions, in particular, in areas, such as attention and memory. Studies confirmed that regular aerobic exercise enhances cognitive functions, such as working memory and attention by strengthening brain plasticity. These benefits mainly stem from neural changes in the brain, such as increased hippocampal volume and improved memory capabilities (Ferrer-Uris et al., 2022; Chaire et al., 2020). The cognitive enhancements linked to exercise, notably, have been validated not only in young adult populations, but also in elderly cohorts, yielding positive outcomes across various age groups (Ferrer-Uris et al., 2022; Chaire et al., 2020).

Besides, exercise enhances the functionality of the prefrontal cortex, hence strengthening individuals’ executive functions, comparising decision-making, problem-solving, and self-control—higher-order cognitive processes. Study has suggested that exercise can improve cognitive functions and emotional management abilities by increasing levels of BDNF and augmenting oxygenation in relevant brain regions (Kukla-Bartoszek and Głombik, 2024). This improvement is crucial for coping with daily life challenges and stressors. Specifically, for individuals with depression, enhanced executive functions strengthen better management of negative emotions and maladaptive behavioral patterns, hence strengthening psychological health. For instance, one study uncovered that increased exercise elevated the activation of the prefrontal cortex, hence enhancing emotional regulation and decision-making abilities (Mullen and Hall, 2015). Furthermore, another study suggested that the relationship between exercise and executive function, particularly, is pronounced in elderly populations, where higher cardiovascular health levels are linked to better executive control performance (Buckley et al., 2014; Zhou and Tolmie, 2024).

Exercise can also serve as a positive adjunct to cognitive-behavioral therapy (CBT), aiding individuals in strengthening cognitive functions and enhancing therapeutic outcomes, hence more positively managing negative emotions and behavioral patterns. Study has uncovered that physical exercise enhances executive functions, in particular, by increasing cognitive flexibility, problem-solving abilities, and the capacity to adapt to new challenges—critical aspects for emotion management and the regulation of negative cognitions (Mullen and Hall, 2015). For example, combining physical exercise with CBT has been suggested to significantly slow anxiety symptoms and improve cognitive functions, hence helping individuals more positively address emotional distress and behavioral issues (Mullen and Hall, 2015).

### 5.2 Emotional regulation and stress management

Exercise promotes emotional stability and reduces emotional fluctuations through multiple mechanisms. Regular exercise enhances the activity of brain regions linked to emotional regulation, such as the prefrontal cortex and the amygdala, which play pivotal roles in emotion control (Lim et al., 2021). For example, one study has uncovered that exercise increases the cortical thickness of the prefrontal cortex, hence enhancing emotional regulation capabilities, especially when confronting negative emotions (Wu et al., 2022). Furthermore, exercise has been suggested to improve the processing of emotions and alleviate depressive symptoms, further strengthening emotional stability (Kukla-Bartoszek and Głombik, 2024).

Taking part in regular exercise helps individuals enhance their stress-coping abilities, reducing the intensity and frequency of stress responses, hence mitigating the negative impact of stress on psychological health. Study confirms a positive correlation between exercise and positive psychological health indicators, and a negative correlation with negative indicators, where exercise indirectly influences mental health by enhancing individuals’ psychological resilience (Lin et al., 2024). Furthermore, long-term participation in exercise can improve stress responses by regulating the HPA axis, facilitating better cortisol regulation, and enabling individuals to recover more swiftly when faced with stress (Moyers and Hagger, 2023; Gosadi, 2024).

Exercise provides individuals with various emotional regulation strategies, comparising the release of endorphins through aerobic exercise to enhance mood, hence positively strengthening emotion management and regulation capabilities. One study has suggested that higher levels of exercise are significantly linked to reductions in depressive and anxiety symptoms, indicating that physical exercise can serve as a positive emotional regulation strategy for managing emotional distress (Zhang et al., 1888). Regular participation in aerobic exercise not only increases the secretion of endorphins, but also reduces stress hormones such as cortisol levels, hence strengthening overall mood and psychological health (Mu et al., 2024). Furthermore, mind–body exercise forms such as yoga and meditation have been confirmed to enhance self-awareness and emotional control abilities, hence helping individuals more positively manage and regulate their emotions, reducing the occurrence of depressive symptoms (Bhatt et al., 2024).

Overall, exercise positively influences emotional stability and psychological health through multiple mechanisms, comparising enhancing the activity of brain regions involved in emotional regulation, strengthening stress responses, and strengthening emotion management capabilities. Consequently, regular participation in exercise is a vital avenue for strengthening emotional stability and psychological wellbeing.

### 5.3 Social interaction and support

Taking part in group physical activities, such as team sports, fitness classes, and yoga sessions, not only provides a conducive platform for physical exercise, but also positively promotes the establishment of social interactions and support networks (Neațu et al., 2024). Group and team sports foster a sense of belonging and provide social support, helping to alleviate feelings of loneliness and lower the incidence of depressive symptoms (Li et al., 2024). For example, group sports can not only lessen the sense of loneliness in individuals with depression, but also strengthen their social support networks (Elgendy et al., 2024). Furthermore, exercise plays a significant role in slowing depressive emotions by enhancing self-concept and increasing social support. Study confirms that physical exercise not only directly affects depressive emotions, but also indirectly reduces depressive levels among university students by strengthening self-concept and increasing social support (Zhang et al., 2022).

Exercise, in particular, team sports, significantly enhances individuals’ social skills and interpersonal communication abilities. Studies have uncovered that sports exercise not only directly improves social adaptability, but also indirectly enhances social adaptability by strengthening social–emotional competence and self-esteem (Liu et al., 2023). Moreover, regular exercise helps improve emotion regulation and social skills, which are essential for establishing and maintaining healthy interpersonal relationships (Martín-Rodríguez et al., 2024). For individuals with depression, participating in sports activities can not only alleviate mental health issues, but also reduce social isolation by enhancing self-confidence and strengthening social interactions, hence strengthening psychological recovery.

Research has suggested that individuals with robust social support networks exhibit better recovery capabilities and lower relapse rates when facing depression. Social support is considered crucial for psychological health, especially in the treatment of depression, where social interactions commonly accompany exercise, helping to improve mood and alleviate depressive symptoms (Blake, 2012). Furthermore, exercise enhances individuals’ social support systems by strengthening social interactions, hence providing significant psychological relief and support. Various types of exercise interventions, such as aerobic exercise and strength training, can positively facilitate individuals’ psychological health, while social support interventions aim to provide social support directly or indirectly, further enhancing individuals’ emotional wellbeing (Snowden et al., 2015).

Overall, group physical activities positively reduce depressive symptoms and improve overall psychological health by strengthening social interactions, enhancing social support, strengthening self-concept, and strengthening emotion regulation capabilities. This suggests that strategies combining exercise with social support hold significant application value in the prevention and treatment of depression.

### 5.4 Improvement of self-efficacy and self-esteem

Self-efficacy refers to an individual’s confidence in their ability to accomplish specific tasks and cope with challenges. Study has suggested that exercise significantly enhances individuals’ self-efficacy by setting and achieving exercise goals, hence positively affecting psychological health (Li et al., 2024). Particularly in adult and elderly populations, exercise slows depressive symptoms by enhancing individuals’ perceptions of endurance, strengthening self-esteem, and strengthening emotional states (Wei et al., 2024). Furthermore, exercise-induced self-efficacy not only regulates emotional states, but also improves interpersonal relationships and self-esteem, helping individuals overcome difficulties, persist in their goals, and enhance their sense of life’s meaning during physical activities, hence further reducing depressive symptoms (Mu et al., 2024).

Participation in exercise significantly elevates individuals’ self-esteem. Studies have uncovered that sports exercise indirectly alleviates depressive symptoms and promotes improvements in psychological health by increasing self-esteem (Wei et al., 2024). Furthermore, the impact of sports exercise on depression is partly mediated by enhancing self-image and positive psychological capital, hence strengthening individuals’ intrinsic motivation to cope with depression. Exercise provides clear opportunities for goal setting and achievement, causing the establishment of a positive self-identity. Study confirms that taking part in exercise enhances psychological health, boosts self-confidence, and increases life satisfaction, hence reducing anxiety and depressive feelings (Wei et al., 2024). Moreover, regular physical exercise helps alleviate depressive symptoms, elevate self-esteem, and facilitate the formation of a positive life attitude (Wei et al., 2024).

Overall, exercise plays a crucial role in preventing and slowing depressive symptoms by enhancing individuals’ self-efficacy and self-esteem through multiple avenues. These improvements in emotional states and interpersonal relationships underscore the importance of integrating exercise into daily life as a fundamental strategy for positive psychological health interventions.

### 5.5 Integration and interaction between physiological and psychological mechanisms

Although physiological and psychological mechanisms have largely been discussed separately, emerging evidence underscores that these two dimensions are inherently interconnected, jointly mediating the beneficial effects of exercise on depressive symptoms. A clearer understanding of how psychological processes interact with physiological changes provides deeper mechanistic insights and has crucial implications for developing comprehensive intervention strategies. While clinical evidence consistently supports exercise as a positive intervention for depression, the biological mechanisms remain partially understood due to the complexity of depression’s neurobiological underpinnings and the multifaceted molecular effects of exercise (Ross et al., 2023). Overcoming these challenges requires a multidisciplinary approach that integrates systems biology with large-scale public health investment to bridge the gap between mechanistic findings and standardized treatment guidelines.

Exercise-induced improvements in cognitive functions, such as memory, executive control, and attention, are closely tied to physiological adaptations in the brain, notably increases in hippocampal volume and enhanced neural plasticity (Ferrer-Uris et al., 2022; Kukla-Bartoszek and Głombik, 2024). Elevated levels of brain-derived neurotrophic factor (BDNF), triggered by exercise, serve as a critical physiological mediator linking increased neurogenesis and neuroplasticity with cognitive enhancement (Jasińska-Mikołajczyk et al., 2022; Murawska-Ciałowicz et al., 2021). This improvement in cognitive functioning further supports psychological resilience, empowering individuals to more positively manage negative thoughts and emotional challenges characteristic of depression (Mullen and Hall, 2015). Such cognitive resilience likely provides sustained benefits, highlighting a synergistic relationship between exercise-induced neural changes and cognitive psychological adaptation.

Besides, emotional regulation and stress-management benefits of exercise, highlighted by improved prefrontal cortex functioning and modulation of the hypothalamic–pituitary–adrenal (HPA) axis (Moyers and Hagger, 2023; Wu et al., 2022), reflect deep interactions between physiological stress responses and psychological coping mechanisms. For example, the exercise-mediated reduction in cortisol secretion—a physiological response—directly correlates with reduced anxiety and enhanced emotional stability—a psychological outcome (Moyers and Hagger, 2023; Gosadi, 2024). Furthermore, psychological resilience fostered through exercise can itself attenuate physiological stress responses, creating a reinforcing feedback loop that progressively enhances individuals’ capacity to cope with stressors (Lin et al., 2024; Mu et al., 2024).

Social interactions facilitated by exercise also demonstrate a clear interplay between physiological and psychological processes. Physiologically, group exercise settings not only elevate endorphins, facilitating positive affect and social bonding (Loeb et al., 2022; Schoenfeld and Swanson, 2021), but also reduce inflammatory markers such as IL-6 and CRP, known to be elevated in conditions of social isolation (Cerqueira et al., 2020; Zheng et al., 2019). Psychologically, increased social interaction and support networks established through group activities foster a sense of belonging, strengthening self-esteem and self-efficacy (Elgendy et al., 2024; Zhang et al., 2022). Thus, physiological reductions in inflammation synergistically interact with psychological increases in social connectedness to comprehensively reduce depressive symptoms.

It is notable that improvements in self-efficacy and self-esteem through physical activity involve closely integrated physiological and psychological pathways. Achieving physical exercise goals triggers dopaminergic reward pathways—physiological mechanisms associated with increased dopamine secretion—which subsequently enhance psychological self-efficacy, motivation, and positive self-concept (Ando et al., 2024; Wei et al., 2024). Additionally, exercise-induced physiological improvements, such as enhanced cardiovascular fitness, translate directly into enhanced physical competence and body image, fostering psychological benefits like increased self-confidence and reduced depressive symptoms (de Souza et al., 2017; Li et al., 2024; Wei et al., 2024). Such integrative relationships underscore the multidimensional nature of exercise’s therapeutic effects on depression.

In summary, these interactive mechanisms suggest that exercise plays antidepressant effects through a dynamic interplay between physiological and psychological pathways rather than through isolated mechanisms alone. Future interventions that strategically target this integrated biopsychological nexus may achieve greater therapeutic efficacy, supporting a more holistic approach to depression management across the lifespan.

### 5.6 Exercise modalities and their differential effects on depression

Different exercise methods involve different physiological and psychological mechanisms, affecting the neurotransmitter system, neural plasticity, inflammatory response, and overall mental health. Understanding the different effects of various forms of exercise is crucial for optimizing treatment strategies for patients with depression. Table 3 displays exercise modalities and their differential effects on depression.

**Table 3:** Exercise modalities and their differential effects on depression.

Exercise modality	Effects on depression	References
Aerobic exercise	Enhances neurotransmitter levels (serotonin, dopamine, norepinephrine), increases BDNF, improves cardiovascular function, reduces systemic inflammation.	Schuch et al. (2018), Liberato et al. (2016)
Resistance training	Improves self-efficacy and self-esteem, increases IGF-1, modulates HPA axis, enhances mood regulation.	Schuch et al. (2016), Collins et al. (2019), Athanasiou et al. (2023)
Adolescents—team sports, dance, aerobic exercise	Enhances neuroplasticity, cognitive function, and emotional resilience, particularly positive due to social engagement.	Samsudin et al. (2024)
Adolescents—HIIT	Stimulates dopamine release, improves stress regulation.	Tyler et al. (2023)
Middle-aged adults—aerobic & resistance training	Reduces stress, improves emotional regulation, beneficial for metabolic syndrome and burnout.	Wang et al. (2025)
Elderly—low-impact aerobic exercise	Maintains cognitive function, prevents neurodegenerative diseases, reduces social isolation.	Yang et al. (2025)
Elderly—tai chi	Improves balance, lowers inflammation, enhances social connectedness, reduces fall risk.	Chen et al. (2023), Kuang et al. (2024)
Treatment-resistant depression—high-intensity aerobic exercise	Modulates neuroinflammatory pathways, restores neurotransmitter balance, reduces microglial proliferation.	Dantzer et al. (2008), Perry and Teeling (2013)
Depression with comorbid anxiety—yoga, tai chi, moderate aerobic exercise	Enhances vagal tone, lowers cortisol levels, improves emotional regulation.	Sani et al. (2023)
Depression with comorbid anxiety—resistance training	Promotes dopamine release, enhances executive function, reduces stress-related depression.	Schuch et al. (2016)

#### 5.6.1 Aerobic exercise vs. resistance training in depression treatment

Aerobic exercise is one of the most extensively studied modalities for treating depression. Numerous meta-analyses and randomized controlled trials (RCTs) have consistently displayed that moderate-to-vigorous aerobic exercise significantly reduces depressive symptoms. This effect is mediated through multiple mechanisms, including the enhancement of neurotransmitter levels (e.g., serotonin, dopamine, norepinephrine), elevation of brain-derived neurotrophic factor (BDNF), improvement in cardiovascular function, and reduction of systemic inflammation (Schuch et al., 2018; Liberato et al., 2016).

In contrast, resistance training has gained increasing attention for its antidepressant effects, particularly among populations with comorbid anxiety, stress-related disorders, and cognitive decline (Schuch et al., 2016). Resistance training has been shown to improve self-efficacy and self-esteem, increase levels of insulin-like growth factor-1 (IGF-1) (Collins et al., 2019), and modulate the hypothalamic–pituitary–adrenal (HPA) axis, leading to enhanced mood regulation (Athanasiou et al., 2023).

#### 5.6.2 Exercise modalities across various age groups

The effects of exercise on depression vary across age groups due to differences in neuroplasticity, hormonal balance, cardiovascular health, and social engagement needs.

Adolescents: Exercise benefits adolescents mainly through enhancing neuroplasticity, cognitive function, and emotional resilience. Given their heightened social sensitivity, team-based sports, dance, and aerobic exercises appear most positive in slowing depressive symptoms (Samsudin et al., 2024). High-intensity interval training (HIIT) has also gained attention as a positive intervention for adolescent depression, potentially due to its ability to stimulate dopamine release and improve stress regulation (Tyler et al., 2023).

Middle-aged adults: This population often experiences depression due to chronic stress, occupational burnout, and metabolic syndrome. Both aerobic and resistance training are beneficial, with mind–body exercises or moderate-intensity resistance training being particularly positive in improving emotional regulation without inducing excessive physical fatigue (Wang et al., 2025).

Elderly individuals: Depression in the elderly is often associated with cognitive decline, reduced mobility, and social isolation. Low-impact aerobic exercises (e.g., brisk walking, swimming, cycling) and resistance training are beneficial for maintaining cognitive function and preventing neurodegenerative diseases (Yang et al., 2025). Tai chi is particularly valuable for elderly individuals, as it improves balance, reduces fall risk, enhances social connectedness, and lowers inflammation—all causes linked to depression prevention in aging populations (Chen et al., 2023; Kuang et al., 2024).

#### 5.6.3 Exercise for specific depression subtypes: treatment-resistant depression and comorbid anxiety

Certain depression subtypes, such as treatment-resistant depression (TRD) and depression with comorbid anxiety, may require specialized exercise interventions. Research indicates that TRD patients often exhibit chronic HPA axis dysregulation and elevated levels of neuroinflammation (Dantzer et al., 2008). High-intensity aerobic exercise (e.g., treadmill running, cycling at 70–80% of maximum heart rate) may be particularly beneficial in modulating neuroinflammatory pathways and restoring neurotransmitter balance (Perry and Teeling, 2013). Aerobic exercise has been recognized as a positive mechanism for preventing and treating various neuroinflammatory conditions, reducing microglial proliferation, reducing the expression of immune-related genes in the hippocampus, and decreasing the expression of inflammatory cytokines such as TNF-α (Dantzer et al., 2008; Perry and Teeling, 2013).

Besides, anxiety disorders commonly co-occur with depression, necessitating exercise interventions that stabilize the autonomic nervous system and reduce excessive sympathetic activation. Yoga, tai chi, and moderate-intensity aerobic exercise have shown efficacy in reducing both depressive and anxious symptoms by enhancing vagal tone, reducing cortisol levels, and improving emotional regulation (Sani et al., 2023). Resistance training may also benefit individuals with stress-related depression and anxiety, as it promotes dopamine release and enhances executive function (Schuch et al., 2016). This comprehensive analysis highlights the differential effects of exercise modalities across various populations and depression subtypes, stressing the importance of tailored interventions to optimize therapeutic outcomes.

#### 5.6.4 Challenges and obstacles of exercise for patients with depression

Although exercise is widely regarded as a positive intervention for treating depression, the relationship between exercise and depression is bidirectional, meaning that depression itself can hinder an individual’s ability to engage in physical activity. Some psychological, physiological, and social disorders cause low compliance and participation in exercise programs among patients with depression. Psychologically speaking, the features of depression are low motivation, lack of pleasure (inability to experience happiness), and negative self perception (Pu et al., 2024). This may make initiating and maintaining exercise programs particularly challenging. Physiologically, chronic fatigue, dysregulated stress responses (elevated cortisol levels), and neurobiological imbalances (such as low dopamine and serotonin activity) further cause decreased energy levels and exercise avoidance progression (Liao and Zhang, 2006). In addition, social withdrawal and isolation—common features of depression—can reduce participation in group or structured exercise programs, especially for those with self-awareness or social anxiety (Stein and Stein, 2008). Recognizing these barriers is crucial for developing positive and personalized exercise interventions. Someone has proposed monitoring plans, such as digital health interventions, to improve exercise adherence in patients with depression (Silverman et al., 2021). Addressing these challenges can not only improve the feasibility of exercise based interventions, but also maximize their long-term positiveness in depression management.

## 6 Comparative analysis of age-dependent mechanisms

The pathogenesis of depression demonstrates significant variations across various age groups, and the physiological and psychological mechanisms by which exercise alleviates depression also differ with age. Identifying these age-dependent mechanistic differences is crucial for progressing targeted intervention strategies. This section compares the distinct physiological and psychological responses among adolescents, middle-aged, and elderly populations to elucidate the unique pathways through which exercise mitigates depression in each age group. Table 4 demonstrates comparative analysis of age-dependent physiological and psychological mechanisms by which physical activity alleviates depression.

**Table 4:** Comparative analysis of age-dependent physiological and psychological mechanisms by which physical activity alleviates depression.

Age group	Physiological mechanisms	Psychological mechanisms	References
Adolescents	Promotes neuroplasticity and neurogenesis in the hippocampus, Enhances expression of BDNF, Regulates hormonal balance (testosterone and estrogen), and Improves cognitive functions and learning abilities.	Enhances self-esteem and social skills, Promotes social interactions through team sports and group activities, Reduces feelings of loneliness and social anxiety, and Improves coping with academic and social pressures.	Hu et al. (2023), Kukla-Bartoszek and Głombik (2024), Azevedo et al. (2020), Feng et al. (2024)
Middle-aged	Restores HPA axis balance, Reduces inflammatory responses, Decreases cortisol secretion, and Mitigates cardiovascular issues and psychological distress linked to chronic stress.	Enhances emotional regulation and stability, Improves stress management capabilities, Provides outlets for emotional release, reducing negative emotions, and Strengthens overall mental health.	Yiallouris et al. (2019), Huang et al. (2013), Al-Wardat et al. (2024), Holland et al. (2024)
Elderly	Increases cerebral blood flow, Delays neurodegenerative changes, Regulates immune function by reducing levels of chronic inflammatory markers (e.g., C-reactive protein [CRP], interleukin-6 [IL-6]), and Strengthens cognitive functions.	Reduces feelings of loneliness, Enhances social support and interactions, Boosts self-efficacy and self-esteem, and Rebuilds confidence through setting and achieving exercise goals, reducing self-doubt and helplessness related to physical decline.	Du and Kong (2024), Abbatecola et al. (2024), Umegaki (2022), Xiong et al. (2023), Li et al. (2023)

### 6.1 Differences in physiological mechanisms

Adolescence is a period defined by rapid neurological progression, during which exercise plays a crucial role in brain maturation and neurotransmitter regulation. Studies have suggested that exercise promotes neuroplasticity, enhances the expression of BDNF, and stimulates neurogenesis in the hippocampus. These changes cause improved cognitive functions and learning abilities (Azevedo et al., 2020). Furthermore, adolescents undergo significant hormonal fluctuations during puberty. Exercise helps regulate hormonal balance, in particular, levels of testosterone and estrogen, hence stabilizing mood and slowing depressive symptoms (Kukla-Bartoszek and Głombik, 2024).

Interestingly, middle-aged individuals commonly face occupational stress, family responsibilities, and physiological changes related to perimenopausal hormonal fluctuations. Chronic stress can cause dysregulation of the HPA axis, affecting the secretion of stress hormones such as cortisol and increasing the risk of depression. Study confirms that lifestyle modifications, especially exercise and stress management, can help restore HPA axis balance, hence strengthening mental health (Yiallouris et al., 2019). Large-scale epidemiological evidence has shown that increased vigorous exercise is associated with an 11% reduction in depression risk per day of activity, whereas prolonged sedentary time is linked to higher depression odds (Huang and Huang, 2023a). These findings highlight the critical role of physical activity in preventing stress-induced depressive symptoms during midlife. Moreover, exercise reduces inflammatory responses and declines cortisol secretion, mitigating cardiovascular issues and psychological distress linked to chronic stress (Huang et al., 2013).

It is worth noting that in the elderly population, physiological mechanisms mainly involve neurodegenerative changes and immune function decline. Exercise enhances cerebral blood flow and promotes neuroplasticity, hence delaying brain structural degeneration and strengthening cognitive functions (Du and Kong, 2024). Studies have displayed that exercise fosters neuroplasticity and improves cerebral blood flow, slowing down age-related brain structural deterioration (Abbatecola et al., 2024). Concurrently, exercise regulates the immune system by reducing levels of chronic inflammatory markers such as CRP and IL-6, hence slowing inflammation-induced mood disorders (Umegaki, 2022).

Overall, exercise slows depressive symptoms through distinct physiological mechanisms tailored to different life stages. During adolescence, exercise promotes neural progression and hormonal regulation (refers to the body’s physiological mechanisms maintaining hormonal balance, involving the regulation of stress hormones (e.g., cortisol) and mood-related hormones [e.g., endorphins, dopamine], which significantly influence emotional stability and psychological wellbeing), mainly enhancing brain function and emotional stability. In middle age, exercise modulates stress hormones and improves cardiovascular health, hence mitigating stress-related depressive symptoms. In older adults, exercise can delay neurodegenerative changes and regulates immune function, mainly preventing and slowing depression linked to physiological decline.

### 6.2 Differences in psychological mechanisms

Adolescents are in a critical stage of self-identity formation and social adaptation, where exercise plays a vital role. Study confirms that sports activities not only positively alleviate depressive and anxiety symptoms in adolescents, but also correlate positively with mental health, strengthening brain health and cognitive functions (Hu et al., 2023). Furthermore, participation in team sports and group activities fosters social interactions, reduces feelings of loneliness and social anxiety, and enhances self-esteem, hence helping adolescents better cope with academic and social pressures (Feng et al., 2024).

Besides, middle-aged individuals mainly alleviate depressive symptoms through emotional regulation and stress management. Studies have suggested that exercise positively impacts emotional regulation and mental health, in particular, in slowing depression and anxiety symptoms. Regular exercise not only helps regulate emotional responses, but also enhances emotional stability and improves stress management capabilities (Al-Wardat et al., 2024). Furthermore, exercise provides outlets for emotional release, helping to discharge accumulated negative emotions and thereby strengthening psychological wellbeing (Holland et al., 2024). By Taking part in physical activities, middle-aged individuals gain physical health benefits and enhance their overall mental health by strengthening their emotional regulation abilities.

Notably, elderly individuals face significant psychological challenges, comparising social isolation and a decline in self-worth. Study has suggested that exercise can significantly reduce feelings of loneliness and enhance social support by strengthening social interactions and building support networks (Xiong et al., 2023). Furthermore, participation in exercise boosts self-efficacy and self-esteem in the elderly. Through setting and achieving exercise goals, older adults can rebuild confidence in their abilities, reducing self-doubt and feelings of helplessness linked to physical decline (Li et al., 2023). In elderly individuals, depression is often exacerbated by chronic diseases, nutritional deficiencies, and systemic inflammation. Studies have shown that diet plays a crucial role in modulating depression risk in older adults. A machine learning-based retrospective cohort study found that dietary intake of potassium, vitamin E, and vitamin K significantly correlated with depressive symptoms, suggesting that nutritional interventions may serve as an adjunct therapy for late-life depression (Huang and Huang, 2023b).

Overall, the psychological mechanisms by which exercise alleviates depression exhibit significant age-dependent differences. Adolescents mainly enhance self-esteem and social skills through exercise, using exercise to improve self-efficacy and social adaptation. Middle-aged individuals mitigate stress-related depressive symptoms through emotional regulation and stress management facilitated by exercise. Elderly individuals reduce loneliness and enhance self-worth by strengthening social interactions and strengthening self-esteem through exercise.

## 7 Methodological considerations and quality assessment of current literature

Despite extensive literature supporting exercise as a viable non-pharmacological intervention for depression across various age groups, methodological quality and rigor among these studies vary considerably, potentially limiting the strength and generalizability of their conclusions. It is crucial to acknowledge these methodological limitations to accurately interpret current findings and guide future research.

### 7.1 Methodological limitations of current research

One prominent methodological limitation is the predominant use of cross-sectional and short-term intervention designs. While these approaches provide valuable preliminary insights, they fail to adequately capture the sustained, long-term effects of exercise interventions on depressive symptoms (Schuch et al., 2016; Jasińska-Mikołajczyk et al., 2022; Wei et al., 2024). Consequently, the durability of beneficial effects over extended periods remains uncertain, potentially restricting the translation of findings into lasting clinical practice. Additionally, many studies exhibit small sample sizes or rely heavily on self-report measures, potentially introducing biases and limiting the robustness and generalizability of conclusions. For example, while studies examining exercise-induced changes in neurotransmitter levels or inflammatory markers frequently utilize objective biomarkers (Murawska-Ciałowicz et al., 2021; Zheng et al., 2019), studies assessing psychological outcomes often rely mainly on self-report questionnaires, increasing the risk of subjective bias (Zhang et al., 2022; Feng et al., 2024). Moreover, there is considerable variability in exercise interventions employed, including differences in type, frequency, duration, and intensity, which complicate comparisons and limit generalizability (Schuch et al., 2016; Cerqueira et al., 2020). Such variability further restricts the strength of evidence regarding optimal exercise prescriptions tailored to specific populations. In addition, studies rarely systematically address potential confounding causes such as participants’ baseline physical fitness, prior exercise history, comorbid medical conditions, and concurrent medication use, all of which significantly influence depressive symptoms and treatment outcomes (Blake, 2012). Furthermore, current literature largely neglects the exploration of individual differences, such as genetic susceptibility, gender-specific physiological responses, and lifestyle variations, potentially obscuring the nuanced positiveness of exercise interventions (Jasińska-Mikołajczyk et al., 2022; Wei et al., 2024).

### 7.2 Recommendations for future methodological enhancements

To address these limitations and enhance methodological rigor in future research, several key considerations should be prioritized: (i) Future studies should adopt longitudinal designs, involving long-term follow-up to investigate the persistence of exercise-related benefits on depression over months or years, thereby clarifying the sustainability of treatment effects (Schuch et al., 2016; Blake, 2012). (ii) Systematic reviews and meta-analyses focusing specifically on methodological rigor are required to critically evaluate the strength of evidence. Employing standardized assessment tools, such as Cochrane Risk of Bias (RoB 2) or GRADE criteria, can assist in identifying high-quality studies and areas where evidence is lacking or inconclusive. (iii) Future research should explicitly standardize exercise protocols—clearly specifying exercise type (e.g., aerobic vs. resistance training), intensity, frequency, duration, and adherence measures—to facilitate comparability across studies. Clear and replicable intervention protocols would significantly enhance reliability and generalizability of findings (Snowden et al., 2015). (iv) Future studies should aim to integrate both objective (e.g., biomarkers, neuroimaging) and subjective measures (e.g., validated psychological scales) in evaluating intervention positiveness. Combining these measures would strengthen the credibility and precision of outcomes, elucidating mechanisms more accurately (Jasińska-Mikołajczyk et al., 2022; Zheng et al., 2019; Kukla-Bartoszek and Głombik, 2024). (v) Longitudinal studies and randomized controlled trials (RCTs) with sufficiently large sample sizes, spanning diverse age groups and involving follow-ups over several years, are needed. Such designs would allow investigation of age-dependent, sustained effects of exercise, facilitating more accurate age-specific guidelines for depression interventions (Ferrer-Uris et al., 2022; Mullen and Hall, 2015). (vi) Research should further investigate individualized and combined intervention approaches, including integration with cognitive-behavioral therapy, mindfulness, and pharmacotherapy. Such multimodal approaches may offer complementary or synergistic effects, potentially amplifying treatment outcomes, particularly in populations resistant to single-treatment modalities (Mullen and Hall, 2015; Bhatt et al., 2024).

## 8 Practical applications and policy implications

### 8.1 Clinical applications and guidelines

Clinicians can utilize insights from age-dependent mechanisms of exercise to tailor interventions more positively. For adolescents, integrating group-based aerobic exercises or team sports into psychological care plans can capitalize on exercise-induced improvements in social skills, cognitive function, and emotional stability, addressing the unique academic and social pressures characteristic of this population (Li et al., 2024; Feng et al., 2024). For middle-aged adults experiencing chronic stress or burnout, combining structured aerobic exercise with mindfulness or stress-management training (e.g., yoga, meditation) may synergistically alleviate depression by modulating cortisol levels and improving emotional regulation (Moyers and Hagger, 2023; Gosadi, 2024). In elderly populations, integrating moderate aerobic activity with cognitive-behavioral interventions or social engagement programs (such as group exercise sessions or community fitness initiatives) can positively mitigate loneliness, enhance cognitive resilience, and slow age-related cognitive decline, thereby improving overall mental health (Mullen and Hall, 2015; Xiong et al., 2023; Li et al., 2023). Clinical recommendations suggest that structured exercise interventions should be tailored based on individual needs, with supervised group sessions improving adherence. A systematic review of exercise therapy for depression highlighted that moderate-intensity aerobic or mind–body exercise (3–5 sessions per week, lasting 4–16 weeks) is positive in reducing depressive symptoms (Xie et al., 2021). The review also emphasized that higher-dose exercise yields better overall functional improvements, further supporting the need for personalized exercise prescriptions.

To facilitate clinical implementation, healthcare practitioners should incorporate personalized assessments, including baseline fitness, comorbidities, and patient preferences, into individualized exercise prescriptions. Routine clinical evaluations could also integrate objective biomarkers (e.g., BDNF, inflammatory cytokines) alongside psychological assessments to monitor treatment efficacy more accurately.

### 8.2 Public health implications

From a public health perspective, evidence presented in this review supports prioritizing exercise as a primary, accessible, and cost-positive intervention for mental health promotion and depression prevention. Public health authorities could enhance population-level mental wellbeing by implementing age-targeted exercise programs in schools, workplaces, and senior community centers. Schools could integrate daily physical activity programs focusing on aerobic exercises and team sports, thereby simultaneously promoting neuroprogressional health, social skills, and resilience among adolescents. Workplace wellness initiatives that encourage regular physical activity, such as flexible scheduling for exercise breaks, provision of fitness facilities, or group fitness programs, could substantially mitigate stress-related depression among middle-aged adults. Similarly, public health campaigns targeting elderly populations should emphasize easily accessible, age-appropriate physical activities (e.g., community walking groups, Tai Chi), highlighting both cognitive and emotional health benefits. In addition, interestingly, from a public health perspective, integrating lifestyle-based interventions—including both physical activity and optimized nutrition—may enhance mental wellbeing and reduce the burden of depression. Recent machine learning-based studies have identified key nutritional causes, such as potassium and vitamin E intake, as significant predictors of depressive symptoms, suggesting that dietary interventions should be considered alongside exercise-based strategies to promote mental health (Huang and Huang, 2023b). Given the strong evidence supporting exercise as a positive strategy for depression prevention, public health policies should prioritize physical activity promotion at a population level. A large-scale U.S. study displayed that each additional day of vigorous exercise significantly reduced depression risk, while increased sedentary behavior was associated with higher depressive symptoms (Huang and Huang, 2023a). These findings emphasize the necessity of integrating exercise into mental health promotion strategies to enhance societal wellbeing.

### 8.3 Policy recommendations

To translate scientific evidence into broader societal impact, policymakers should incorporate exercise interventions into mental health strategies and national health agendas explicitly. Policies could include increased funding and infrastructural support for community-based exercise programs targeting vulnerable populations, integrating physical activity guidelines into mental health care standards, and incentivizing healthcare providers to prescribe exercise interventions alongside or instead of pharmacological treatments where appropriate.

Moreover, educational policies might benefit from requiring structured physical activity programs in schools, specifically designed to enhance psychological resilience and mental health literacy from adolescence. Policymakers should also consider investing in infrastructure and resources (e.g., public parks, accessible fitness centers, digital platforms promoting virtual or home-based exercise) to facilitate regular physical activity at the population level.

### 8.4 Policy recommendations

Based on the comprehensive evidence reviewed, we recommend that health policymakers prioritize: (i) Incorporate exercise interventions explicitly within mental healthcare guidelines, recommending standardized, evidence-based exercise regimens tailored to specific age groups and clinical contexts. (ii) Allocate public funds toward creating community exercise facilities, parks, and affordable fitness programs, thereby reducing socioeconomic barriers and promoting equitable access to mental health interventions. (iii) Encourage healthcare systems to develop integrated mental health programs combining exercise with psychological treatments, such as cognitive-behavioral therapy (CBT) or mindfulness-based stress reduction (MBSR), enhancing treatment efficacy through complementary mechanisms.

### 8.5 Policy recommendations

To translate scientific insights into actionable policy, we recommend that governments and health authorities explicitly recognize exercise as a front-line intervention for depression in national mental health policies. Specifically, policies should support: (i) Establishing national guidelines on physical activity prescriptions for mental health. (ii) Encouraging insurance coverage for clinically prescribed exercise interventions. (iii) Promoting interdisciplinary collaboration among psychologists, psychiatrists, physiologists, exercise scientists, and policymakers to develop comprehensive and integrative mental health strategies. Such integrative policy initiatives could markedly enhance public awareness, adherence, and acceptance of exercise-based interventions as a crucial component in depression prevention and treatment.

## 9 Conclusions and future directions

### 9.1 Summary of key findings

Our review has comprehensively explored the physiological and psychological mechanisms by which exercise alleviates depression across various age groups. The findings indicate that exercise positively mitigates depressive symptoms through various physiological pathways (such as neurotransmitter regulation, hormonal balance, and modulation of inflammation and immune functions) and psychological pathways (comparising cognitive function enhancement, emotional regulation, social support augmentation, and improvement of self-efficacy and self-esteem). The differences in physiological and psychological mechanisms across age groups underpin the age-dependent features of exercise in the treatment of depression. Specifically, during adolescence, exercise promotes neural progression and regulates pubertal hormones, hence enhancing brain function and emotional stability; in middle age, exercise modulates stress hormones and improves cardiovascular health, slowing stress-related depressive symptoms; and in older adults, exercise delays neurodegenerative changes and regulates immune function, preventing and mitigating depression linked to physiological decline.

### 9.2 Practical implications

Developing targeted intervention strategies based on the mechanistic differences in how exercise alleviates depression across various age groups holds significant practical importance: (i) Adolescents: Encourage participation in diverse physical activities, in particular, team sports and aerobic exercises, to strengthen neural progression and enhance social skills. Schools and families should provide supportive environments to reduce academic and social pressures, hence strengthening self-esteem and self-efficacy. (ii) Middle-aged individuals: Center on stress reduction and emotional management by strengthening comprehensive exercise programs, comparising aerobic exercises, strength training, and yoga. These activities help middle-aged individuals regulate stress hormone levels and improve cardiovascular health. Furthermore, workplaces and families should offer increased psychological support to facilitate work-life balance. (iii) Elderly individuals: Advocate for low-intensity, moderate physical activities such as walking, Tai Chi, and aquatic exercises to delay cognitive decline, enhance immune function, and facilitate social interactions. Communities and healthcare institutions should provide accessible exercise facilities and social activities to reduce social isolation, hence enhancing self-esteem and life satisfaction among the elderly.

### 9.3 Future research directions

Despite substantial progress in elucidating the mechanisms by which exercise alleviates depression, several critical gaps remain in current literature, warranting further exploration: (i) Although evidence increasingly supports exercise as an positive intervention for depression, there is limited research examining the combined positiveness of exercise with other non-pharmacological therapies, such as cognitive-behavioral therapy (CBT), mindfulness-based stress reduction (MBSR), or other psychotherapeutic modalities. Future studies should investigate the synergistic effects of these combined approaches, potentially revealing more positive, multifaceted strategies for treating depression across different populations. (ii) While individual physiological and psychological mechanisms of exercise have been extensively studied, research that comprehensively explores the interaction among these pathways remains scarce. Future work should focus on understanding how physiological mechanisms (e.g., neurotransmitter changes, hormonal regulation, immune modulation) interact with psychological processes (such as cognitive improvement, emotional regulation, and enhanced self-efficacy) to produce holistic mental health benefits. Using advanced neuroimaging, molecular biomarkers, and computational modeling approaches could significantly advance understanding in this domain. (iii) Current literature often lacks long-term follow-up, with most research limited to relatively brief intervention periods. Longitudinal studies across multiple life stages could better capture the sustained impact of exercise on depressive symptoms, mental health resilience, and overall quality of life. Conducting comparative longitudinal studies across adolescents, adults, and elderly populations would provide robust insights into the temporal dynamics and differential effects of exercise interventions at various progressional stages, informing targeted public health policies and clinical practices. (iv) Research to date has inadequately addressed individual variability, including differences in gender, genetic background, lifestyle, socioeconomic status, and pre-existing health conditions. Future work should prioritize developing personalized exercise interventions tailored to these individual causes. By incorporating precision medicine approaches and employing predictive analytics based on biomarkers such as BDNF, cortisol levels, and inflammatory cytokines, interventions could achieve higher efficacy, adherence, and patient outcomes. (v) The emergence of digital technologies, including virtual reality (VR), wearable fitness trackers, mobile health applications, and tele-exercise platforms, presents significant opportunities for enhancing participation, adherence, and outcomes of exercise interventions for depression. Future research should explore how technology-assisted interventions can overcome barriers to participation, especially among elderly or geographically isolated individuals, thereby expanding accessibility and improving mental health outcomes across diverse populations.
